# Climate Change and Sustainability in Health Professions Education: A Realist Review Protocol

**DOI:** 10.12688/hrbopenres.14282.1

**Published:** 2025-11-18

**Authors:** Marah Elfghi, Emer Galvin, Deirdre Bennett, Niamh Coakley, Deborah Heaphy, Rory Mulcaire, Caoimhe O'Brien, Claudia Osborne, Anél Wiese

**Affiliations:** 1 Medical Education Unit, University College Cork School of Medicine, Cork, County Cork, T12 AK54, Ireland

**Keywords:** Climate change, sustainability, planetary health, health professions education, realist review

## Abstract

**Background:**

Climate change poses a critical global health challenge, affecting public health, healthcare systems, and health professions education (HPE). While healthcare professionals play a key role in addressing climate-related health risks and promoting sustainable practices, formal training in climate change and sustainability (CC&S) remains limited and CC&S education is inconsistently implemented due to challenges such as curriculum constraints and lack of faculty expertise. Existing systematic and scoping reviews provide an overview of CC&S interventions but do not sufficiently explore the mechanisms driving their success or failure. A realist review is needed to understand what works, for whom, and under what conditions in CC&S education.

**Methods:**

This realist review will follow the RAMESES publication standards and use a structured, iterative approach to synthesise evidence. A comprehensive search strategy will be conducted across academic databases and grey literature sources to identify CC&S education interventions in undergraduate, postgraduate, and continuing professional education across healthcare disciplines. Data will be extracted using a Context-Mechanism-Outcome (CMO) framework to analyse key contextual factors, mechanisms, and outcomes influencing intervention effectiveness. Regular team discussions will ensure consensus in identifying CMOs and refining the initial programme theory. Findings will be reported through narrative synthesis, summary tables, and a graphical representation of the final programme theory.

**Discussion:**

This review will provide practical insights for stakeholders on how to effectively integrate CC&S education into HPE curricula. By unpacking mechanisms and contextual factors, it will go beyond traditional systematic reviews to explain why and how these interventions succeed or fail. The findings will inform curriculum development, faculty training, and policy recommendations, ensuring that future healthcare professionals are equipped to address climate-related health challenges and advance sustainable healthcare practices. Ultimately, this review will contribute to the global effort to embed sustainability within HPE, preparing the healthcare workforce for a changing climate.

Systematic review registration: Open Science Framework (OSF)

## Background

Climate change is a critical global health challenge with profound implications for public health, healthcare systems, and health professions education (HPE) ^1^. Rising temperatures, extreme weather, shifting disease patterns, and ecosystem disruptions are worsening disease burdens and increasing health risks worldwide ^2^. Beyond impacting public health, climate change also threatens healthcare system accessibility and sustainability ^3^. Moreover, the healthcare sector contributes 4–5% of global greenhouse gas emissions through energy use, waste, and the carbon footprint of medical supply chains ^4^. This underscores the urgent need to integrate sustainability into healthcare delivery and education to mitigate climate change impacts while ensuring high-quality, resilient healthcare systems.

Healthcare professionals play a critical role in addressing the health consequences of climate change and promoting sustainable healthcare practices ^5^. As frontline providers, they must be equipped with the knowledge, skills, and attitudes to recognise climate-related health impacts and integrate sustainability into clinical practice that support both environmental and human health ^6, 7^. However, despite growing recognition of these responsibilities, formal training in CC&S remains limited in HPE ^8– 10^. Surveys among healthcare students and practitioners indicate that while interest in CC&S education is high, opportunities for structured learning remain limited ^9, 11– 15^. This gap has led to calls for the systematic integration of CC&S education into HPE programmes globally ^16– 19^.

International organisations, professional bodies, policymakers and educational institutions have advocated for embedding CC&S education into HPE. The Lancet Countdown on Health and Climate Change ^20^, the Global Consortium on Climate and Health Education ^21^, and Health Care Without Harm ^22^ emphasise the urgent need to equip healthcare professionals with climate and sustainability competencies to enhance preparedness and response efforts. Similarly, national accreditation bodies—such as the UK General Medical Council ^23^, the Association of American Medical Colleges ^24^, and The Royal College of Physicians and Surgeons of Canada ^25^ — stress the health sector’s responsibility in climate adaptation and mitigation. The Global Climate and Health Alliance ^26^ further reinforces the need for targeted education programmes, while the International Federation of Medical Students’ Associations ^27^ has played a key role in student-led advocacy for climate-conscious medical curricula. Together, these initiatives highlight the critical need for CC&S education in HPE to ensure that future healthcare professionals are adequately prepared to address the challenges posed by climate change.

Despite this growing recognition of CC&S education, implementation remains inconsistent across different health professions, educational institutions, and geographic regions ^16, 28^. Several barriers have hindered the systematic integration of CC&S into HPE curricula. Many educators lack formal training or expertise in CC&S, making it difficult to incorporate these topics into exiting curricula ^29, 30^. Health professions programmes are densely packed with clinical, biomedical, and technical content, making it challenging to introduce additional CC&S topics without restructuring existing curricula ^29– 31^. Without clear curriculum guidelines or accreditation requirements, CC&S education remain optional rather than an essential competency ^10, 32^. While various teaching approaches (e.g., case-based learning, online modules, simulations, workshops) have been used to introduce CC&S education, there is insufficient research on which methods are most effective across different learning contexts ^28^. Many CC&S initiatives in HPE rely on short-term funding or voluntary efforts limiting their scalability and long-term impact.

Three recent scoping reviews highlight the current landscape of CC&S education in HPE. The first review explored nursing students' perspectives and evaluated various teaching strategies for integrating CC&S into nursing curricula ^33^. The second review examined interprofessional education on climate change and health, identifying a limited number of studies with diverse intervention designs, underscoring the need for further research and standardised approaches to interprofessional CC&S education ^34^. A third scoping review mapped the global integration CC&S content in undergraduate medical education found that most implementations began between 2020 and 2021, using stand-alone modules, electives, and curriculum modifications, with student-led initiatives playing a key role ^31^. A systematic review on sustainable healthcare education in HPE found that various interventions, including workshops and clinical skills sessions, effectively improved learners' knowledge, attitudes, and skills, though no single approach was superior, and challenges such as limited curriculum time and faculty expertise hindered broader implementation ^28^.

Systematic reviews often focus on the effectiveness of interventions without considering the mechanisms underlying their success or failure. Whereas scoping review methods provide descriptive overviews. These methods rarely consider contextual variations – such as institutional policies, faculty engagement, or student motivation – that shape the effectiveness of education. In contrast, realist reviews aim to unpack the underlying mechanisms and contextual factors that shape outcomes, offering a deeper understanding of why and how CC&S education programmes succeed or fail ^35^. Realist reviews have been successfully applied in HPE to examine diverse areas, including workplace learning ^36^, online medical education ^37^, and remediation programmes ^38^. However, to our knowledge, no realist review has systematically examined the implementation and effectiveness of CC&S education interventions for healthcare professionals across different contexts.

The guiding research question for this review is: "What works, for whom, and under what circumstances in climate change and sustainability education in health professions education?" This review will examine CC&S education interventions across undergraduate, postgraduate, and continuing professional education, identifying the key contextual factors, mechanisms, and outcomes that shape their effectiveness. It will consider both formal curriculum-based interventions and extracurricular or professional development initiatives, offering a comprehensive understanding of best practices in CC&S education. The scope will span diverse healthcare disciplines, including medicine, nursing, and allied health professions.

Guided by learning ^39, 40^, behavioural ^41, 42^, and implementation science theories ^43^, this review will identify the mechanisms driving CC&S education interventions. A core outcome of this realist review will be the development of a programme theory, providing a structured, theoretical framework to enhance the understanding and implementation of CC&S education in HPE.

By synthesising evidence through a realist lens, we aim to generate practical insights that can guide the design and implementation of impactful and sustainable educational strategies. This protocol outlines the methodology for our realist review, detailing the scope, objectives, and approach to data collection and analysis. Ultimately, our findings will contribute to the growing body of knowledge on sustainability education and support the integration of climate-conscious practices in HPE.

## Methods

A realist review will be conducted, following the RAMESES Publication Standards for Realist Syntheses ^44^ and associated training materials ^45^. Realist principles will be integrated into all stages of the review process. This review protocol follows the guidelines outlined in PRISMA-P checklist ^46^, *Appendix 1*.

This realist review will follow a structured process to analyse CC&S education interventions by examining context, mechanisms, and outcomes. First, we will refine the research question and establish the review scope. Next, we will develop an initial programme theory, outlining how CC&S education is expected to work. A comprehensive literature search will then be conducted across relevant databases and grey literature sources. Identified studies will undergo systematic screening and selection based on detailed selection criteria. The analysis and synthesis phase will apply realist principles to extract and identify patterns in CMO configurations, refining the programme theory iteratively. Finally, the refined programme theory will be presented in narrative, tabular, and graphical formats, providing actionable insights for educators, policymakers, and institutions to support the effective integration of CC&S education in health professions curricula.

### Defining the scope of the review

This realist review will focus on CC&S education interventions across various formats—including online, in-person, workplace, classroom, and group-based settings—targeting healthcare professionals and students from disciplines such as medicine, nursing, dentistry, pharmacy, and allied health. By including studies from undergraduate, postgraduate, and continuing professional development contexts, the review aims to capture a comprehensive picture of how CC&S education is being implemented and evaluated globally. The scope is further defined to incorporate empirical research—both published journal articles and relevant grey literature—that assesses intervention effectiveness, using quantitative, qualitative, or mixed-method approaches. This focus and these boundaries ensure that the review concentrates on interventions directly impacting educational practices and outcomes, while excluding non-empirical work, studies outside healthcare, non-educational interventions, and theoretical papers without practical implementation. This deliberate framing facilitates a nuanced understanding of what works, for whom, and under what conditions in CC&S education within HPE, thereby providing actionable insights for curriculum design and policy development.

### Initial Programme Theory

The Initial Programme Theory (IPT) for this realist review was developed through an iterative process, integrating existing literature, curricular documents, and theoretical frameworks. The IPT serves as the starting framework for the review, outlining how CC&S education interventions are expected to work, for whom, and under what conditions.

A structured theoretical approach guided the development of the IPT, focusing on three key dimensions necessary for embedding CC&S education: (1) behavioural change, (2) effective learning, and (3) institutional integration.

To address behavioural change, the COM-B Model was selected for its focus on how Capability, Opportunity, and Motivation interact to shape learners' ability to engage with CC&S practices ^42^. Additionally, the Theory of Planned Behaviour explains how attitudes, social norms, and perceived behavioural control influence whether learners adopt CC&S behaviours in their professional practice ^41^.

For effective learning, multiple theories were incorporated. Social Constructivist Learning Theory emphasises collaborative learning, experiential knowledge-building, and social interactions, all of which are critical for CC&S education. Transformative Learning Theory highlights how learners undergo perspective shifts through critical reflection and real-world engagement ^40^, that could lead to long-term commitment to CC&S principles. Sociocultural Learning Theory emphasise learning takes place through engagement within a social context, encompassing both formal education (structured, intentional instruction) and informal learning (implicit understanding of norms and practices within a given setting) ^39^. It underscores that individuals are part of a larger social whole, where learning occurs through active participation in meaningful activities. In CC&S education, this means not just acquiring knowledge but continuously shaping and being shaped by one’s professional and environmental context.

For institutional integration, Normalisation Process Theory ^43^ can be used to examine how CC&S education can become embedded in healthcare curricula and professional practices, focusing on processes of adoption, implementation, and sustainability. The Consolidated Framework for Implementation Research ^47^ provides a structured way to assess barriers and facilitators at multiple levels, including organisational culture, leadership engagement, and policy alignment.

A key step in developing the IPT involved reviewing curricular documents from HPE to identify intended learning outcomes, pedagogical approaches, and competency frameworks related to CC&S education ^48^. This helped establish potential contexts for CC&S education, such as interprofessional learning environments, problem-based learning approaches, and experiential simulations. Additionally, it outlined anticipated outcomes, including enhanced sustainability literacy, clinical decision-making informed by planetary health principles, and advocacy for sustainable healthcare practices.

This refined IPT will guide the analysis of evidence, ensuring that CMO configurations are systematically explored to understand what works, for whom, and under what conditions in CC&S education.

### Search strategy

For this review, an initial comprehensive electronic literature search will be conducted across relevant databases, including Academic Search Complete, Australian Education Index, British Education Index, Business Source Complete, CINAHL, Embase, ERIC, PsycINFO, PubMed, Scopus, and SocINDEX, as well as grey literature sources. The search will target literature discussing climate change and sustainability interventions aimed at enhancing health professions education.

The systematic search strategy is built around three keyword groups: (1) healthcare professionals, (2) climate change and sustainability, and (3) education interventions, linked using Boolean operators (OR and AND). Wherever possible, a combination of free-text terms and Medical Subject Headings (MeSH) terms will be used to optimise search sensitivity. The final search strategy is detailed in *Appendix 2*, with adaptations made for different databases to ensure transparency and reproducibility. Searches will be restricted to titles and abstracts, and the process may involve multiple rounds of searching to refine and investigate specific areas of interest in greater detail.

Additionally, backward and forward citation tracking will be employed to enhance the comprehensiveness of the review. Backward citation tracking will be done by checking reference lists of selected articles and forward citation tracking will be done by using citation indexes such as Scopus and Web of Science to identify more recent studies that have cited key articles, ensuring the inclusion of the latest research developments in CC&S education in HPE.

### Selection criteria

Inclusion criteria:

•    Studies reporting on CC&S educational interventions in any format, e.g. online, in-person, workplace, classroom, small group, large group, any duration, etc.,

•    Studies reporting on health professionals and students from a range of health disciplines, such as medical doctors, nurses, dentists, pharmacists, dietitians, dispensing opticians, medical scientists, occupational therapists, optometrists, physical therapists, physiotherapists, podiatrists, chiropodists, radiographers, radiation therapists, speech and language therapists and clinical biochemists. Various levels of training (e.g., undergraduate, postgraduate, CPD) will be included,

•    Studies reporting on evaluation of the intervention,

•    Studies that are empirical journal articles or grey literature containing empirical data,

•    Quantitative, qualitative, and mixed-method studies,

•    Studies published between January 2014 and October 2024,

•    Studies published in English.

Exclusion criteria:

•    Non-empirical studies including commentaries, letters, editorials, and reviews,

•    Studies not related to healthcare students or professionals.

•    Non-educational interventions (e.g., clinical interventions, policy interventions)

•    Papers reporting on educational frameworks or curriculums without implementing an intervention.

### Selection process

Studies identified through the database search will be exported to Covidence for screening. Duplicate records will be removed automatically by Covidence and manually verified by one reviewer. The title and abstract screening will be conducted independently by two reviewers, who will regularly meet with the research team to discuss progress and resolve any uncertainties. If disagreements arise, a third reviewer will be consulted to make a final decision. Articles will be excluded if they do not meet the inclusion criteria.

During screening, reviewers will have access to bibliographic fields, including title, abstract, authors, journal name, and year of publication, and will not be blinded to these details. For full-text screening, the research team will first conduct a pilot screening with a sample of studies to ensure consistency. Full-text screening will also be performed by two independent reviewers, who will be blinded to each other’s decisions.

If full-text access is unavailable, the research team will attempt to contact the corresponding authors via email. These communications will remain confidential, and no metadata will be shared. However, efforts to obtain full texts will be documented, including the number of authors contacted and the outcomes of these requests.

This screening process balances methodological rigour with researcher resources, ensuring a systematic and transparent selection of studies while maintaining consistency and reliability in the inclusion process.

### Data extraction

A structured data extraction process will be implemented to systematically collect study characteristics, intervention details, evaluation methods, and Context-Mechanism-Outcome (CMO) configurations. Data extraction will be conducted using two tools: a Microsoft Excel spreadsheet to document general study characteristics and a CMO Data Extraction Template specifically designed to identify and record CMO configurations.

The Excel spreadsheet will capture key study characteristics, including title, author(s), year of publication, study type, a description of the intervention (including format, duration, and delivery method), details on how the intervention was evaluated, and the population involved (e.g., healthcare discipline, student or professional level).

In parallel, the CMO Data Extraction Template will focus on identifying mechanisms and contextual factors influencing the effectiveness of CC&S education interventions. This template consists of two primary components: a Contextual Information Table, which captures key details such as the target population and educational format used (e.g., online, in-person, workshop, integrated curriculum), and a CMO Table, where explicitly stated or inferred CMO configurations are recorded, providing insight into how specific contexts influence mechanisms to produce particular outcomes.

Additionally, the template includes a quality and relevance assessment section, where reviewers will evaluate the strengths and limitations of each study, assess its richness in identifying CMO configurations, and determine its usefulness in refining the programme theory. To ensure rigour and reliability, two independent reviewers will extract data from each study. Regular team discussions will be conducted to review and refine the CMOs, resolve any disagreements regarding what constitutes a CMO, and ensure a systematic approach to data extraction. This iterative process will help refine the programme theory, ensuring that the evidence collected contributes meaningfully to understanding what works, for whom, and under what conditions in CC&S education interventions.

### Analyses and synthesis

The analysis and synthesis process will involve systematically reviewing all Context-Mechanism-Outcome configurations extracted from individual studies to identify patterns and regularities across the dataset and to identify common mechanisms, the contexts in which they operate, and the outcomes they produce. This process will be iterative, allowing for continuous refinement of the initial programme theory as new insights emerge.

The refined programme theory will be developed based on the evidence, integrating key findings from the literature to enhance its explanatory power. Results will be reported in a narrative synthesis, supported by summarised data in table format to present key contextual information, mechanisms, and outcomes in a structured manner. Additionally, a graphic representation of the final programme theory will be created to visually illustrate the relationships between contexts, mechanisms, and outcomes, providing a clear and accessible framework for understanding the findings.

## Study status

At the time of publication, the review is undergoing full-text screening.

## Discussion

This realist review protocol outlines a structured approach to examining CC&S education in HPE. Given the growing recognition of climate change as a global health crisis and the need for sustainability education in healthcare, this review aims to bridge the gap between existing interventions and their effectiveness across different contexts.

By systematically extracting and synthesising Context-Mechanism-Outcome configurations, this review will contribute to evidence-based curriculum development, guiding educators, policymakers, and institutions in designing effective and sustainable CC&S education strategies. The findings will support the integration of sustainability principles into healthcare education, ensuring that future healthcare professionals are equipped to address climate-related health challenges and advocate for environmentally responsible practices. Ultimately, this realist review will provide actionable insights for embedding CC&S education into HPE curricula globally, strengthening the healthcare sector’s capacity to contribute to climate adaptation and mitigation efforts.

## List of abbreviations

**Table:** 

**CC&S**	Climate change and sustainability
**CMO**	Context-Mechanism-Outcome
**HPE**	Health professions education
**IPT**	Initial Programme Theory
**MeSH**	Medical Subject Headings

## Declarations

### Ethics approval and consent to participate

Not applicable

### Consent for publication

Not applicable

## Data Availability

Open Science Framework. Climate Change and Sustainability Interventions in Health Professions Education: A realist review protocol. https://osf.io/4u97s/ ^49^. This project contains the following underlying data: Realist review protocol extended data.pdf (Includes the PRISMA-P Checklist and the Final Search Strategy as supplementary materials supporting the realist review protocol). Data is available under the terms of the Creative Commons Attribution 4.0 International Public License.
