# Supplementary material for: Climate Change and Sustainability in Health Professions Education: A Realist Review Protocol
Source: HRB Open Res. 2026 Feb 2;8:122. Originally published 2025 Nov 18. [Version 2] doi: 10.12688/hrbopenres.14282.2 (PMC12891960; doi:10.12688/hrbopenres.14282.2)
Supplement: Peer review history

# Reviewer report: Reviewer response for version 2

**DOI:** 10.21956/hrbopenres.15797.r53257

**Published:** 2026-02-10

**Authors:** Edlyne Eze Anugwom

**Affiliations:** 1 University of Nigeria, Nsukka, Nigeria

The review has taken care of the comments in my earlier submission.

 Therefore, the paper is now okay and acceptable.

Is the study design appropriate for the research question?

Yes

Is the rationale for, and objectives of, the study clearly described?

Yes

Are sufficient details of the methods provided to allow replication by others?

Partly

Are the datasets clearly presented in a useable and accessible format?

Not applicable

Reviewer Expertise:

NA

I confirm that I have read this submission and believe that I have an appropriate level of expertise to confirm that it is of an acceptable scientific standard.

# Reviewer report: Reviewer response for version 1

**DOI:** 10.21956/hrbopenres.15713.r52299

**Published:** 2026-01-06

**Authors:** Michelle McLean

**Affiliations:** 1 Bond University, Gold Coast, Australia

The article is clearly written and for the most, I know what the authors want to achieve.

 I don't believe I had access to the two appendices. 

 A few considerations: 

 P3, second column, Line 4. should read 'CC&S education remains..' (currently 'remain'). Same line 'an essential competency' should read 'a set oc competencies' as there are many competencies in different domains relating to CC&S. 

 Same page and column, paragraph starting with "Systematic reviews... " The second 'sentence' starting "Whereas... " is not a sentence. 

 Same paragraph, the sentence should read "To the best of our knowledge, no ...".

 Page 4, Column 1, paragraph beginning "Guided by learning.." near the top of the page, "programme theory" is used. I am not sure what to suggest as an alternative, but programme theory is not an informative term. A programme is usually the complete degree and all of its content and activities. 

 Page 4, Column 1, paragraph beginning "This realist.." What is meant by 'mechanism'? For me, a mechanism involves 'how'. Is it a delivery methods, a format, an educational intervention? 

 Selection criteria

 1.Although other disciplines have been excluded, and including them will be impossible, it is probably worth including a note to acknowledge that, in terms of the interrelated triple planetary crisis of a changing climate, biodiversity loss and pollution, solutions are going to come from many different fields, not just health.

 2. Much water has flowed under the bridge since October 2024 so the review will be outdated by the time it is published. I think that for the review to be current, the end date should be kept open until the article is about to be published. This has happened to me on three occasions this year. The review process has taken so long that I have had to add an addendum or replace references. For example, in June this year, a seventh planetary boundary was crossed (ocean acidification). 

 Page 6, Column 1, Paragraph 2, starting "In parallel, ..", "educational format" includes what (i.e. activity, e.g. assignment, lecture, etc.) and how (in teams, face-to-face, etc.) . 

 List of abbreviations

 CMO. Outcome. This could be short-term, e.g. learner satisfaction, which is the most common form of outcome as the longer-term outcomes such as competencies in the workplace, are difficult to measure. Longitudinal studies are required with multi-source feedback. Perhaps 'outcome' needs to be clarified.

Is the study design appropriate for the research question?

Yes

Is the rationale for, and objectives of, the study clearly described?

Yes

Are sufficient details of the methods provided to allow replication by others?

Yes

Are the datasets clearly presented in a useable and accessible format?

Not applicable

Reviewer Expertise:

Medical education, including evaluation and assessment, curriculum development and planetary health.

I confirm that I have read this submission and believe that I have an appropriate level of expertise to confirm that it is of an acceptable scientific standard.

# Response

**Published:** 2026-01-22

**Authors:** Marah Elfghi

**Affiliations:** Medical Education, University College Cork School of Medicine, Cork, County Cork, Ireland

Dear Reviewer,

 We would like to sincerely thank you for your careful reading of our manuscript and for your constructive and detailed feedback. We appreciate your positive assessment of the clarity of the article and the helpful suggestions provided. Below, we respond to each of your comments and outline the revisions made to the manuscript.

**Access to Appendices**

**1.Comment:** The appendices were not accessible.

**Response:**

 Thank you for bringing this to our attention. The appendices are provided as supplementary material in Reference 49

**Editorial and Language Corrections:**

**2.Comment:**

- “CC&S education remain” should read “remains”.
- “An essential competency” should read “a set of competencies”.
- The sentence beginning “Whereas…” is incomplete.
- “To our knowledge” should read “To the best of our knowledge”
**Response:**

We thank the reviewer for noting these issues.

 All suggested grammatical and stylistic corrections have been implemented, including:

- Correction of verb agreement (“remains”) (pg.4)
- Revision to “a set of competencies” to reflect the multidimensional nature of CC&S competencies (pg.4)
- Correction of the incomplete sentence to ensure grammatical completeness (pg.5)
- Revision to “To the best of our knowledge” for academic precision (pg.5)

**Use of the Term “Programme Theory”**

**3.Comment:** The term “programme theory” may be misleading, as “programme” is often interpreted as a full degree programme.

**Response:**

 We appreciate this important conceptual clarification. We have revised the manuscript to explicitly define “programme theory” in the realist sense, clarifying that it refers to an explanatory theory of how educational *interventions* work, rather than an entire degree programme. Additional explanatory text has been added at first use to avoid ambiguity and support readers less familiar with realist methodology. (pg. 5)

**Clarification of “Mechanism”**

**4.Comment:** The meaning of “mechanism” is unclear and may be confused with delivery methods or formats.

**Response:**

 We agree with this observation. We have added a clear definition of “mechanism” consistent with realist methodology, explaining that mechanisms refer to the underlying processes (e.g. reasoning, motivation, engagement) triggered by an intervention within a specific context, rather than the intervention activities or delivery formats themselves.

 This clarification explicitly distinguishes mechanisms from teaching methods, formats, or educational activities. (pg. 6)

**Selection Criteria – Scope and Disciplinary Boundaries**

**5.Comment:** Acknowledgement is needed that solutions to the triple planetary crisis extend beyond health disciplines.

**Response:**

 Thank you for this valuable suggestion. We have added a statement acknowledging that addressing the interconnected challenges of climate change, biodiversity loss, and pollution requires contributions from multiple disciplines beyond health.

 We clarify that the present review focuses on health professions education while recognising the importance of broader interdisciplinary action. (pg. 9)

**Search End Date and Currency of the Review**

**6.Comment:** The fixed end date (October 2024) risks the review being outdated by publication.

**Response:**

 We appreciate this practical and important point. We have revised the protocol to state that the search will start from January 2024 onwards. (pg.9)

**Clarification of “Educational Format”**

**7.Comment:** “Educational format” should clarify both the activity and mode of delivery.

**Response:**

 Thank you for this suggestion. We have expanded the definition of “educational format” to explicitly include both the type of activity (e.g. lecture, assignment, workshop, simulation) and the mode of delivery (e.g. individual or team-based, face-to-face or online). (pg.10)

**Clarification of “Outcome” in CMO Framework**

**8.Comment:** Outcomes can be short-term or long-term and should be clarified.

**Response:**

 We agree with you. We have clarified this distinction in the protocol. Short-term outcomes (e.g., knowledge, attitudes, immediate skills acquisition) and long-term outcomes (e.g., sustained practice change, clinical outcomes) will be specified in our outcome definitions. We will include evaluation studies that measure outcomes at different timepoints, and what outcomes we extract will depend on how individual studies were designed and what they measured. Longitudinal studies will be particularly valuable for capturing long-term outcomes. We acknowledge this temporal distinction is important for understanding how mechanisms may operate differently over time. (pg.10)

 We thank the reviewer once again for their thoughtful and constructive feedback. The suggested revisions have significantly improved the precision, clarity, and methodological transparency of the manuscript. We believe the revised version addresses all concerns raised and strengthens the overall contribution of the protocol. We welcome any further guidance you may wish to provide.

 Kind regards,

 Marah****

# Reviewer report: Reviewer response for version 1

**DOI:** 10.21956/hrbopenres.15713.r51720

**Published:** 2026-01-02

**Authors:** Edlyne Eze Anugwom

**Affiliations:** 1 University of Nigeria, Nsukka, Nigeria

**General Observations:**

 The study is as timely as it is also seemingly overdue given the importance of climate change and its repercussions on all spheres of life in contemporary society. There is arguing the fact (despite the existence of pockets of incurable nay-sayers) that climate change is perhaps the most remarkable challenge of life in contemporary society as we know it. It does really begs the question why health professions education has not mainstreamed climate change into its curricular and models of practice soon enough! In this sense, the present study is a timely and welcome intervention that would hopefully energize not only the discourse of climate change in HPE but the evolvement of workable strategies and theory that would guide education and practice. The above notwithstanding, the following observations may be pertinent: 

- The study shows a very good knowledge of the extant literature in the area of focus and this commendable and portrays the fact that the authors are very capable of achieving the aims of the study.
- In addition to the above, the study uses available or extant literature to provide further insights for its goals/outcomes generally. In other words, the authors clearly demonstrate that the study is anchored on evident paucity in the extant literature especially gaps existing in available empirical studies or reviews in the literature.
- In addition, it also drew insights from advocacy and the standing of health policy bodies/accreditation agencies in underlining the overarching need for CCS in HPE.
- However, there are too many jargons and health-related concepts that can potentially limit the value of the study for non-health professionals. Given the nature of climate change as both inter and multi-disciplinary, there may be need to simplify these jargons and go for words/phrases that are within the easy comprehension of general readers.
- Despite the fact that it has become standard fare to talk about ‘realist’ review, the study needs to capture the essence of the so-called realist review i.e., is it different from systematic review or is it part of systematic review. This is important in the light of the fact that there seems some ambivalence in the extant literature regarding the real status of the so-called realist review. For instance, there is the perception of realist review as simply another form of systematic review (see, Pawson et al., 2025 Ref 1) or as the elongation of the usual systematic review to include the why and where questions often glossed over in traditional systematic reviews. Thus, the extant literature would seem to suggest that realist review is an elongation of the systematic review in the sense of going beyond what works to also include for whom and under what circumstances or why (see, Weiss and Bucuvalas, 1980 Ref 2; Pawson, 2006 Ref 3; Wong et al., 2012 Ref 4).
- There is mention of the Context-Mechanism-Outcome (CMO) framework and this seems quite interesting; however, there is need to make explicit what ‘mechanism’ means. In other words, there is need to properly conceptualize it along the lines of what was done with regards to ‘context’.

**Comments on Methods:**

 The authors have done quite an impressive job of detailing and specifying the methods to be adopted in the study. However, I believe that the methods could be further strengthened by looking at the following minor issues:

- There is need to unravel or explain exactly some key phrases used in the protocol, e.g., ‘detailed selection criteria’ – what does this mean? Also, the idea of grey literature though an old practice in qualitative studies should be further elaborated – what is grey literature in this case?
- In addition to the above, the choice of studies or literature published between 2014 and 2024 in the inclusion criteria needs to be justified; why this period? Also, why the exclusion of non-empirical works/studies? This seems strange for a protocol focused on developing a new theory!
- There is the stated objective of the protocol to bridge the gap between existing interventions and their effectiveness across different contexts. As interesting as the above sounds, the critical issue is how this goal can be achieved through the development of a new programme theory. In other words, there is need to further adumbrate or show in clear terms how a new programme theory would bridge this gap or at the minimum the envisaged properties of the theory that could tackle this gap.

**Observations on Goals/Outcomes:**

 The goals/outcomes of the study seem at the same time laudable/impressive and over-ambitious. It would make sense for the researchers to have a clear focus on what is achievable and practicable and not enticed by the desire to solve all problems or cover all universe. Therefore, the following may be helpful:

- The goal of the review protocol to examine CCS in HPE at the undergraduate, postgraduate and even continuing professional education as well as in diverse health care disciplines seems over-ambitious and may lead to the generation or development of a protocol or guide too-unwieldy and over-reaching to provide realistic guidance. It would seem too-much like a one-size-fits-all approach and thus have limited effectiveness/impact.
- There is still need to relate the outcome of the study which is stated to be ‘the development of a programme theory’ to the methodology chosen. A review may yield evidence or results regarding paucity of existing theoretical orientations but how to transit from this paucity (or even gaps) to the evolvement/generation of a new theory needs further nuancing especially since no efforts addressing the critique and evaluation of existing theories were mentioned in the methods. All the same, one must acknowledge the impressive array of theories mentioned that would inform the Initial Programme Theory (IPT); however, these theories are so diverse in both focus and disciplinary orientations that the challenge of how to really and productively synthesize them emerge. And this was not creatively factored into the protocol. Thus, the validity of the envisaged Refined Programme Theory (RPT) may be limited especially as it is expected to apply to a diverse academic, practice and disciplinary worlds. Moreso, there is need to understand the pre-requisites of a good theory generally and the efforts towards critiquing or generating a new theory must abide with some of these major conventions (see, Okeibunor and Anugwom, 2002 Ref 5; Whetten, 1989 Ref 6; Guest, 2024 Ref 7; Lange et al., 2025 Ref 8).  
- Also, the goal to provide actionable insights for embedding CC&S education into HPE curricula globally was mentioned – this seems too ambitious and clearly not feasible given structural, curricula and contextual differences in HPE across the globe. HPE curricular differs across nations and sometimes within countries and regions. Therefore, aiming for actionable insights applicable globally may in truth seem like an overreach. 

References:

- Guest, O. (2024). “What Makes a good Theory, and How Do We Make a Theory Good”? *Computational Brain and Behaviour* 7 (1). https://doi.org/10.1007/s42113-023-00193-2
- Lange, J., Freyer, N., Musfeld, P., Schonbrodt, F., & Leising, D. (2025). “A Checklist for Incentivizing and Facilitating Good Theory Building”. *Zeitschrift fur Psychologie* 233 (4): 279 – 283. https://doi.org/10.1027/2151-2604/a000604
- Okeibunor, J.C. & Anugwom, E.E. (2002). *Sociological Theory: an insight into dominant viewpoints.* Nsukka: Fulladu
- Pawson, R. (2006). *Evidence Based Policy: A Realist Perspective*. London: Sage. https://doi.org/10.1080/15588740802262039
- Pawson, R., Greenhalgh, T., Harvey, G., & Walshe, K. (2025). “Realist Review: A New Method of Systematic Review Designed for Complex Policy Interventions”. *Journal of Health Services Research and Policy* 10 (1): 21 – 34. https://doi.org/10.1258/1355819054308530
- Weiss, C.H., & Bucuvalas, M.J. (1980). *Social Science Research and Decision Making*. Newbury Park: Sage
- Whetten, D. (1989). “What Constitutes a Theoretical Contribution”? *Academy of Management Review* 14 (4): 490 – 495. https://doi.org/10.2307/258554
- Wong, G., Greenhalgh, T., Westhorp G., Pawson, R. (2012). “Realist Methods in Medical Education Research: What are They and What can They Contribute”? *Medical Education* 46 (1):89–96. https://doi.org/10.111/j.1365-2923.2011.04045.x.

Is the study design appropriate for the research question?

Yes

Is the rationale for, and objectives of, the study clearly described?

Yes

Are sufficient details of the methods provided to allow replication by others?

Partly

Are the datasets clearly presented in a useable and accessible format?

Not applicable

Reviewer Expertise:

Social Dimensions of Climate Change; Development Studies; Political Economy; Public Health

I confirm that I have read this submission and believe that I have an appropriate level of expertise to confirm that it is of an acceptable scientific standard, however I have significant reservations, as outlined above.

## References

[1] : Realist review - a new method of systematic review designed for complex policy interventions. *Journal of Health Services Research & Policy* .2005;10(1_suppl) : 10.1258/1355819054308530 21-34 10.1258/1355819054308530 16053581

[2] : Social Science Research and Decision Making. *https://archive.org/details/socialscienceres0000weis* .1980;

[3] : Evidence-Based Policy: A Realist Perspective, by Ray Pawson. *Journal of Policy Practice* .2008;7(4) : 10.1080/15588740802262039 321-323 10.1080/15588740802262039

[4] : Realist methods in medical education research: what are they and what can they contribute?. *Medical Education* .2012;46(1) : 10.1111/j.1365-2923.2011.04045.x 89-96 10.1111/j.1365-2923.2011.04045.x 22150200

[5] : Sociological Theory: an insight into dominant viewpoints.2002;

[6] : What Constitutes a Theoretical Contribution?. *The Academy of Management Review* .1989;14(4) : 10.2307/258554 10.2307/258554

[7] : What Makes a Good Theory, and How Do We Make a Theory Good?. *Computational Brain & Behavior* .2024;7(4) : 10.1007/s42113-023-00193-2 508-522 10.1007/s42113-023-00193-2 PMC1329865642459536

[8] : A Checklist for Incentivizing and Facilitating Good Theory Building. *Zeitschrift für Psychologie* .2025;233(4) : 10.1027/2151-2604/a000604 279-283 10.1027/2151-2604/a000604

# Response

**Published:** 2026-01-22

**Authors:** Marah Elfghi

**Affiliations:** Medical Education, University College Cork School of Medicine, Cork, County Cork, Ireland

Dear Reviewer,

 We sincerely thank you for your thoughtful, generous, and constructive review of our realist review protocol. We greatly appreciate your recognition of the timeliness, relevance, and scholarly grounding of the study, as well as your detailed feedback, which has helped us strengthen the clarity, methodological rigour, and theoretical coherence of the protocol. Below, we respond to each of your comments in turn and outline the revisions made to the manuscript.

**General Observations:**

**1.Comment:** The topic is timely and important, but the manuscript contains excessive jargon that may limit accessibility for non-health professionals.

**Response:**

 We thank the reviewer for this thoughtful observation and agree that climate change is inherently interdisciplinary and of relevance beyond the health sector. The primary intended audience for this realist review, however, is educators, researchers, and policymakers working within health professions education. As such, the use of established terminology from health education, implementation science, and realist methodology is necessary to ensure conceptual precision and alignment with existing scholarly discourse in this field.

 That said, we have carefully reviewed the manuscript to ensure that key technical terms are used consistently and meaningfully, and that their application is appropriate to the aims of the study. Where specialist terminology is essential, it reflects commonly accepted language within health professions education and realist synthesis and is required to maintain methodological clarity and rigour. We believe that further simplification beyond this point would risk diluting important conceptual distinctions central to the realist approach.

**2.Comment:** The manuscript needs to more clearly explain what distinguishes a realist review from a systematic review.

**Response:**

 Thank you for highlighting this important point. We have expanded the Background section to explicitly clarify the epistemological and methodological distinction between realist reviews and traditional systematic reviews. In particular, we now explain that realist reviews are theory-driven and focus on understanding *how*, *why*, *for whom*, and *under what circumstances* interventions work, rather than solely assessing effectiveness.

 We have added text positioning realist review as an extension beyond traditional systematic reviews, drawing on established realist literature to clarify this distinction and address ambivalence in the field. (pg. 5)

**3.Comment:** The Context–Mechanism–Outcome (CMO) framework is mentioned, but the concept of “mechanism” requires clearer conceptualisation.

**Response:**

 We fully agree. We have added an explicit definition and conceptual explanation of “mechanism” within the realist tradition, distinguishing mechanisms from programme activities and outcomes. This clarification is now included in the Methods sections, ensuring consistency and conceptual transparency. (pg. 6)

**Comments on Methods**

**4.Comment:** Key phrases such as “detailed selection criteria” and “grey literature” require clarification.

**Response:**

 Thank you for your comment. We agree with you, we have defined detailed selection criteria: as the predefined inclusion and exclusion conditions used to decide which studies are eligible to be included. (pg.6)

 Additionally, we have expanded the description of grey literature, specifying what sources are included (e.g., policy reports, curricular documents, organisational reports) and explaining their relevance to realist theory development. (pg.8)

**5.Comment:** The inclusion of studies published between 2014 and 2024 requires justification, and the exclusion of non-empirical studies seems contradictory for theory development.

**Response:**

 We appreciate this important critique. We have now justified the 2014 onwards timeframe, noting that most CC&S education initiatives in HPE emerged during this period alongside growing global policy and curricular momentum. (pg.9)

 Regarding non-empirical studies, we have clarified that while theoretical papers are excluded from formal inclusion, theory-informed insights are incorporated during the development of the Initial Programme Theory. Empirical studies are prioritised to ensure that theory refinement is grounded in observed contexts, mechanisms, and outcomes rather than abstract conceptualisation alone. (pg.9)

**6.Comment:** It is unclear how the development of a programme theory will bridge the gap between interventions and their effectiveness across contexts.

**Response:**

 Thank you for your comment. Programme theory bridges this gap by explaining how and why interventions work differently across contexts. It identifies the mechanisms that are activated in particular contexts to produce outcomes, thereby explaining variation in effectiveness through context-mechanism-outcome configurations (as explained in pg.10) rather than simply documenting it.

**Observations on Goals and Outcomes**

**7.Comment:** The goals of the study appear over-ambitious, particularly the inclusion of multiple educational levels and disciplines.

**Response:**

 We acknowledge this concern and have revised the manuscript accordingly. We have refined the stated goals to emphasise theory-building rather than universal prescription, clarifying that the intent is not to propose a one-size-fits-all model but to develop an explanatory framework sensitive to contextual variation across educational levels and disciplines. (pg.5 and pg.11)

**8.Comment:** The relationship between theory development and the realist methodology requires clearer articulation.

**Response:**

 We agree. We have strengthened the linkage between realist methodology and programme theory development, explicitly explaining how iterative CMO analysis leads to theory refinement.

 Furthermore, we have clarified how existing theories inform; but do not predetermine. These frameworks will be selectively refined the programme theory, and we have acknowledged the methodological challenge of synthesising diverse theoretical perspectives. (pg.8 and pg.11)

**9.Comment:** The ambition to generate globally applicable actionable insights is unrealistic given curricular and contextual variation.

**Response:**

 We appreciate this important point. We have revised the language, we now emphasise that findings will offer transferable principles and context-sensitive insights, allowing educators and institutions to adapt recommendations to their local curricular, cultural, and policy environments. (pg.3 and pg.11)

 Once again, we sincerely thank you for your insightful and rigorous review. Your feedback has significantly strengthened the conceptual clarity, methodological transparency, and scholarly contribution of this protocol. We believe the revisions address your concerns and improve the overall quality and utility of the manuscript. We would be happy to address any further comments if required.

 Kind regards,

 Marah
